# Longitudinal anti-SARS-CoV-2 antibody immune response in acute and convalescent patients

**DOI:** 10.3389/fcimb.2023.1239700

**Published:** 2023-09-08

**Authors:** Horacio Pérez-Juárez, Angélica Serrano-Vázquez, Héctor Godínez-Alvarez, Enrique González, Liliana Rojas-Velázquez, Patricia Moran, Tobías Portillo-Bobadilla, Manuel Ramiro, Eric Hernández, Clara Lau, Marcela Martínez, Ma. de los Ángeles Padilla, Martha E. Zaragoza, Blanca Taboada, Laura A. Palomares, Susana López, Alejandro Alagón, Carlos F. Arias, Cecilia Ximénez

**Affiliations:** ^1^ Laboratorio de Inmunología, Unidad de Investigación en Medicina Experimental, Facultad de Medicina, Universidad Nacional Autónoma de México, Mexico City, Mexico; ^2^ Estancias Posdoctorales por México-Consejo Nacional de Humanidades, Ciencias y Tecnologías (CONAHCyT), Mexico City, Mexico; ^3^ Unidad de Biotecnología y Prototipos, Facultad de Estudios Superiores Iztacala, Universidad Nacional Autónoma de México, Mexico City, Mexico State, Mexico; ^4^ Red de Apoyo a la Investigación, Universidad Nacional Autónoma de México, Mexico City, Mexico; ^5^ División de Estudios de Posgrado, Facultad de Medicina, Universidad Nacional Autónoma de Mexico, Mexico City, Mexico; ^6^ Laboratorios de Análisis Clínicos e Imagenología, Biomédica de Referencia, S.A.P.I. DE C.V., Mexico City, Mexico; ^7^ Instituto de Biotecnología, Universidad Nacional Autónoma de México, Cuernavaca Morelos, Mexico

**Keywords:** COVID-19, disease severity, ELISA test, IgM, IgG

## Abstract

Despite global efforts to assess the early response and persistence of SARS-CoV-2 antibodies in patients infected with or recovered from COVID-19, our understanding of the factors affecting its dynamics remains limited. This work aimed to evaluate the early and convalescent immunity of outpatients infected with SARS-CoV-2 and to determine the factors that affect the dynamics and persistence of the IgM and IgG antibody response. Seropositivity of volunteers from Mexico City and the State of Mexico, Mexico, was evaluated by ELISA using the recombinant receptor-binding domain (RBD) of the SARS-CoV-2 Spike protein for 90 days, at different time points (1, 15, 45, 60, and 90 days) after molecular diagnosis (RT-qPCR). Gender, age range, body mass index (BMI), comorbidities, and clinical spectrum of disease were analyzed to determine associations with the dynamics of anti-SARS-CoV-2 antibodies. On 90 days post-infection, individuals with moderate and asymptomatic disease presented the lowest levels of IgM, while for IgG, at the same time, the highest levels occurred with mild and moderate disease. The IgM and IgG levels were related to the clinical spectrum of disease, BMI, and the presence/absence of comorbidities through regression trees. The results suggest that the dynamics of anti-SARS-CoV-2 IgM and IgG antibodies in outpatients could be influenced by the clinical spectrum of the disease. In addition, the persistence of antibodies against SARS-CoV-2 could be related to the clinical spectrum of the disease, BMI, and the presence/absence of comorbidities.

## 1 Introduction

Since December 2019, the world has gone through one of the most significant catastrophic events that humanity has suffered with the appearance of a new respiratory infectious disease, namely “COVID-19” caused by SARS-CoV-2. This disease has affected millions of people’s health and quality of life and has increased mortality worldwide (Samuel et al., 2020; Tsang et al., 2021). Until March 17, 2023, approximately 676,609,955 cases had been recorded and 6,881,955 confirmed deaths from COVID-19 worldwide (Johns Hopkins University and Medicine, 2023). In Mexico, the first case was reported in February 2020, and since then, it has continued to spread among the population (7,483,444 cumulative COVID-19 cases and 333,188 deaths have been reported until now (Johns Hopkins University and Medicine, 2023). Unlike other respiratory viruses, people infected with the SARS-CoV-2 virus may develop severe and even fulminant symptoms after an incubation period of approximately 4 to 14 days (Adil et al., 2021; Lamers and Haagmans, 2022).

Despite the alarming numbers of cases and deaths, most of the population infected with SARS-CoV-2 had mild to moderate symptoms that did not require hospitalization, and even in many cases, they did not have any clinical manifestations associated with the infection (Salian et al., 2021). This variation in clinical manifestations might be related to several factors, including age, gender, obesity, smoking, and comorbid chronic conditions such as hypertension and type 2 diabetes mellitus (Wu et al., 2020; Adil et al., 2021).

Several studies have demonstrated serum antibody responses to SARS-CoV-2 in severe and mild patients who have recovered and are convalescing (Liu et al., 2021; Ni et al., 2021; Tsuchiya et al., 2023). Besides, the seroconversion of IgM and IgG isotypes had no significant differences between critical and non-critical patients (Shang et al., 2020). However, when comparing symptomatic and asymptomatic patients, a reduction in the levels of these antibodies was found in the asymptomatic patients (Long et al., 2020).

Other studies have also shown that the humoral antibody response has a time range of 0 to 10 days and 7 to 14 days for IgM and IgG seroconversion, respectively (Long et al., 2020; Cervia et al., 2021; Takita et al., 2022). Regarding the persistence of antibodies, the IgM and IgG isotypes can be present up to 30 and 120 days after infection, respectively (Rodda et al., 2021; Yamayoshi et al., 2021; Takamatsu et al., 2022). However, the IgG antibodies can also be present up to 5 months or more after infection (Wajnberg et al., 2020; Zhang et al., 2021; Yang et al., 2022; Amellal et al., 2023; Sejdic et al., 2023). In addition, some works reported a positive correlation between the severity of the disease and the high levels of anti-SARS-CoV-2 serum antibodies (Chen et al., 2020; Lynch et al., 2020; Zhao et al., 2020; Glück et al., 2021; Sejdic et al., 2023). However, other studies don’t find an association between disease severity and IgG and IgM antibody titers (Phipps et al., 2020; Murchu et al., 2021; Noda et al., 2021).

Although several immunological studies of patients with COVID-19 have been published, few studies have dealt with the factors affecting the early antibody response and persistence of antibodies in people infected or recovered from COVID-19. The present study aimed to evaluate the early and convalescent serum antibody response of patients infected by SARS-CoV-2 and assess the factors that affect its magnitude and persistence over time.

## 2 Materials and methods

### 2.1 Study design and ethical considerations

This study is a longitudinal study of volunteers from the State of Mexico and Mexico City with COVID-19 diagnosed with a positive RT-qPCR, with or without symptoms, who did not require hospitalization; and a group of healthy volunteers without symptoms and a negative RT-qPCR. None of these groups was immunized against the SARS-CoV-2 virus. The protocol and the consent letter were approved by the Scientific and Ethics Committee of the Medical School of the National University of Mexico (UNAM; FM/DI/034/2020). The Official Mexican Standard NOM-012-SSA3-2007 governed the scientific project involving humans and experimental animals. The Norm includes the compromises assumed in the Helsinki Treaty. Written informed consent was obtained from all volunteers after providing them with detailed information about the study and the sampling procedures, the voluntary nature of participation, and the freeness of diagnostic studies.

Peripheral blood samples were taken from a total of 73 volunteers (45 volunteers from the case group and 28 volunteers from the control group) at different periods after study inclusion (1, 15, 45, 60, and 90 days).

### 2.2 Study population characteristics

The volunteers were classified into two groups: (1) patients with COVID-19 detected by RT-qPCR that did not require hospitalization (i.e., cases group), and (2) clinically healthy individuals without diseases that were not suspected of being infected with COVID-19 and with a negative RT-qPCR test for SARS-CoV-2 (i.e., control group). For each volunteer, we obtained data on sex, age, weight, comorbidities, and signs and symptoms related to COVID-19 infection. We also calculated the body mass index (BMI) which was used to measure the relationship between weight and height, to identify normal weight, overweight, and obesity in volunteers. The patients with COVID-19, in turn, were classified into (a) patients without symptoms (asymptomatic group), (b) patients with mild disease and symptoms of the upper respiratory tract, besides fever, fatigue, myalgia, cough, and runny nose, with no data for pneumonia and SpO2 ≥ 94% and (c) patients with a moderate disease that had pneumonia, frequent fever, dry cough followed by productive cough, and sometimes dyspnea, with or without data of altered oxygen saturation (SpO2 ≥ 90%). The grouping of patients into asymptomatic, mild disease, and moderate disease was carried out under the clinical criteria of the responsible physician (Specialist in Internal Medicine) and considered the clinical guidelines for the treatment of COVID-19 in Mexico (Gobierno de México, 2021).

All characteristics of the population were reported as frequencies and percentages for categorical variables. All variables were analyzed using descriptive statistics (mean, median, standard deviation, minimum and maximum values) to describe the central tendency and the degree of variability in the characteristics of the volunteers.

### 2.3 Evaluation of anti-SARS-CoV-2 antibodies (IgG and IgM) by ELISA

The IgM and IgG anti-SARS-CoV-2 antibody levels for the cases and control groups were determined by ELISA. The receptor binding domain (RBD) of the SARS-CoV-2 (Wuhuan strain) spike protein produced in eukaryotic cells (BHK, HEK, and CHO) was used as the target. This recombinant protein was produced at the Instituto de Biotecnología (IBt), UNAM.

For the ELISA, we followed the instructions described by Stadlbauer et al. (2020) with some modifications. Briefly, 96-well plates were coated with 50 μL of a 2 μg/mL solution of (SARS-CoV-2 RBD protein) in phosphate-buffered saline (PBS). The plates were incubated at 4˚C overnight. The serum samples were heat inactivated in a water bath (56˚ C) for one h and stored at 4˚C until use. Coated plates were washed three times with PBS-T (0.1% Tween-20 in PBS). Four hundred μL of blocking solution [3% bovine serum albumin (BSA) in PBS-T] were added to each well, and the plates were incubated at room temperature (RT) for one h. Control and patient samples were diluted 1:50, and 100 μL of the prepared dilution was transferred to the ELISA plates after removing the blocking solution. After one h incubation at RT, the plates were washed three times with PBS-T. Anti-human IgG or anti-human IgM (γ or µ chain-specific, Sigma A6029, A 6907 respectively) horseradish peroxidase (HRP)-labeled secondary antibody, diluted 1:3000 in 1% PBS-T were prepared, 50 μl of secondary antibody solution was added to each well, and the plate was incubated at RT for one h. After washing, 50 μL of HRP substrate solution [10 mL of 0.1 M citrate buffer pH 4.5 with 10 mg ο;-phenylenediamine (Sigma Chemical Co., St Louis, Mo.) and 4 µL of 30% H202] were added for 10 min, and the enzymatic reaction was stopped by adding 200 μL of 1M H2SO4. The plates were read in a plate reader (Biokinetic Reader-Biotek Instruments) at an absorbance of 490 nm.

### 2.4 ELISA-test validation and determination of the cut-off value

To assess the quality of the ELISA, we used 44 serum samples from clinically healthy adult volunteers with a negative PCR result for SARS-CoV-2, who had not had the disease previously as negative controls.

The optimum cut-off value, like the sensitivity and the specificity for anti-SARS-CoV-2 IgG, and IgM antibodies were determined using a receiver operating characteristic curve (ROC) by taking true positive and true negative serum samples (Morán et al., 2007). The group of true positives was represented by serum samples from 31 volunteers infected with COVID-19, with positive RT-qPCR for the SARS-CoV-2 virus. The control group consisted of the same 44 negative serum samples used to validate the test.

Henceforth, all results of the ELISA test were expressed as an index of positivity (IP), which was determined considering the optimum cut-off point that the ROC curves yielded for anti-SARS-CoV-2 IgG or IgM antibodies (IP= OD sample/cut-off point).

### 2.5 Cycle threshold analysis

The cycle threshold (Ct) values were determined by RT-qPCR in Biomedica de Referencia SA de CV. The SARS-CoV-2 RT-qPCR test provides real-time quantification by first reverse transcribing SARS-CoV-2 RNA into cDNA (RT step) and then performing qPCR, during which a fluorescence signal increases proportionally to the amount of amplified nucleic acid, enabling accurate quantitation of the RNA in the sample (Tom and Mina, 2020). Many qPCR assays involve a Ct cut-off 34 to consider the test positive, allowing the detection of very few starting RNA molecules (Cruz-Rangel et al., 2022).

To estimate whether there was a correlation between Ct values and IP for both IgG and IgM, 31 SARS-CoV-2 PCR-positive serum samples were tested. All the data were analyzed with Pearson’s correlation analysis. The correlation analysis was performed using the JMP statistical software, version 16 (SAS Institute Inc, 2000).

### 2.6 Longitudinal follow-up analysis

To evaluate differences in anti-SARS-CoV-2 IgM and IgG antibody levels over time, depending on the characteristics of the population, serum samples from 73 volunteers were used (45 volunteers from the case group and 28 volunteers from the control group) on 1, 15, 45, 60, and 90 days were used (197 serum samples from the case group: 42 on day 1, 43 on day 15, 41 on day 45, 41 on day 60, and 30 on day 90; and 120 serum samples from the control group: 28 on day 1, 28 on day 15, 24 on day 45, 23 on day 60 and 17 on day 90). Anti-SARS-CoV-2 IgM and IgG antibody levels for all samples were determined by ELISA and the results were expressed as IP. The characteristics of the population evaluated were sex (male and female), age (< 60 and ≥ 60 years), BMI (normal weight: ≤ 25, and overweight and obesity: > 25), comorbidities (with or without comorbidities), and clinical spectrum of disease (asymptomatic, mild, or moderate). The IP was compared between the cases and control groups for IgG and IgM with repeated measures ANOVA, with characteristics of the population as between-subjects and characteristics of population and time as within-subjects. The repeated measures design was used considering that the data were obtained from a longitudinal study to determine if the amount of IgM and IgG was affected by the characteristics of the population and if the amount of these immunoglobulins increased or decreased over time. The IP was log-transformed to meet the assumptions of normality and equality of variances of the ANOVA. A test of orthogonal contrasts was used to determine which means were significantly different between the clinical spectrum of disease and the control group using the R software package. The repeated measures ANOVA and the test of orthogonal contrast were performed using the JMP statistical software, version 16 (SAS Institute Inc, 2018).

### 2.7 Relationship between anti-SARS-CoV-2 IgM and IgG antibody levels and the characteristics of the population

In addition to the repeated measures ANOVA, a regression tree analysis was performed to explore the relationship between the IP of anti-SARS-CoV-2 IgM and IgG antibodies and the characteristics of the study population. The regression tree analysis was independently performed for IgG and IgM on 1 and 90 days. The regression tree analysis was performed in R, version 4.2.0 (R Core Team, 2022) using the rpart software (Therneau et al., 2022).

## 3 Results

### 3.1 Study population characteristics

The study population included 73 volunteers from Mexico City and the State of Mexico. Of this total, 45 individuals (61.6%) were infected with SARS-CoV-2, with or without symptoms, and did not require hospitalization, and 28 individuals (38.4%) were clinically healthy individuals without a previous history of COVID-19.

In the group of cases, 62.2% were female, and 37.8% were male. The age ranged between 12 and 83 years. The BMI varied between 18.37 and 32.46, with a mean of 27.50. The individuals had a spectrum of diseases that went from asymptomatic (17.8%) to mild (15.5%) and moderate disease (66.7%). In addition, 37.8% had other comorbidities, including diabetes, hypertension, allergies, chronic obstructive pulmonary disease, and others (**Table 1**).

**Table 1:** Descriptive characteristics of the study population are grouped by cases (i.e., individuals infected with SARS-CoV-2; n = 45) and control (i.e., healthy individuals; n = 28).

	Characteristics			
	Sex			
	Female		Male	
	n (%)			
**Cases**	28 (62.2)		17 (37.8)	
**Control**	10 (35.7)		18 (64.3)	
	Age			
	Mean	Median	Min	Max
	(± SD)			
**Cases**	49.33 ± 16.93	48	12	83
**Control**	47.54 ± 15.72	52	13	76
	Body mass index (BMI)			
	Mean	Median	Min	Max
	(± SD)			
**Cases**	27.50 ± 2.94	25.97	18.37	32.46
**Control**	24.71 ± 3.17	25.05	18.40	29.94
	Comorbidities			
	Yes		Not	
	n (%)			
**Cases**	17 (37.8)		28 (62.2)	
**Control**	5 (17.9)		23 (82.1)	
	Clinical spectrum of disease (COVID-19)			
	Asymptomatic	Mild	Moderate	Healthy
	n (%)			
**Cases**	8 (17.8)	7 (15.5)	30 (66.7)	–
**Control**	–	–	–	28 (100)

In the control group, 64.3% were male, and 35.7% were female. The age ranged from 13 to 76 years. The BMI varied between 18.40 and 29.94, with a mean of 24.71. Only 17.9% of the individuals had some comorbidities that included diabetes type 2, hypertension, allergies, and others. (**Table 1**). None of the individuals in the control group presented symptoms suggestive of COVID-19 during the entire study.

### 3.2 Validation of immuno-assays (ELISA test) and determination of cut-off value

**Figure 1:**
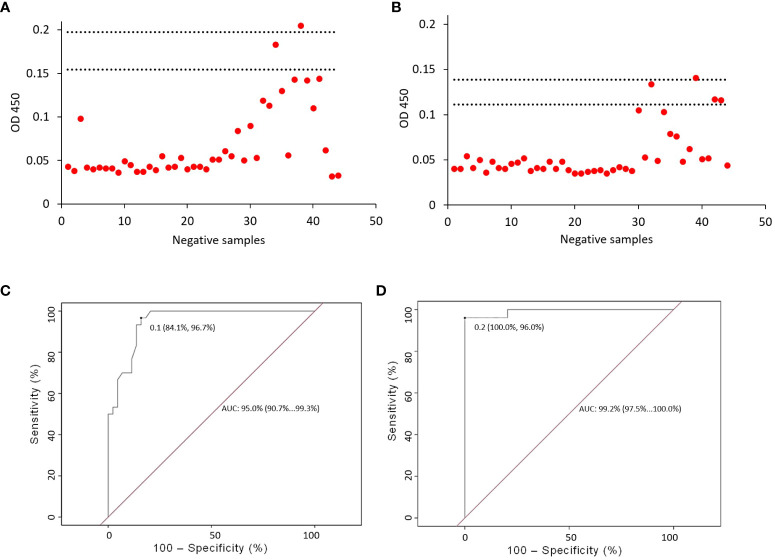
ELISA validation. The validation of the ELISA assays was carried out with 44 serum samples from patients negative for SARS-CoV-2, at a dilution of 1:50. Two and three standard deviations (lower and upper horizontal lines, respectively) were established for anti-SARS-CoV-2 IgM **(A)** and IgG **(B)**. For IgM isotype **(A)**, we obtained a cut-off value of 0.16 which included 2 SD (mean = 0.07, 2 SD = 0.09). When we included 3 SD, the cut-off value was 0.20 (mean = 0.07, 3 SD = 0.13). For IgG isotype **(B)**, we obtained a cut-off value of 0.12 that included 2 SD (mean = 0.06 2 SD = 0.06). When we added 3 SD, the cut-off was 0.15 (mean = 0.06, 3 SD = 0.09). Determination of the cut-off value and evaluation of the sensitivity and specificity of the ELISA. The optimal cut-off value for anti-SARS-CoV-2 IgG and IgM antibodies was determined using a receiver operating characteristic curve (ROC). The curve (ROC) allowed us to obtain the cut-off value for IgM, which was set at 0.1 **(C)**, the sensitivity calculated for IgM was 84.1% and the specificity was 96.7%. For IgG, the cut-off value was set at 0.2 **(D)**, the calculated sensitivity was 100.0% and the specificity was 96.0%.

To validate the ELISA system, samples with no history of SARS-CoV-2 were used as a negative control. A mean of 0.07 (± 0.04) was obtained for IgM and 0.06 (± 0.03) for IgG. In the case of IgM, we obtained a cut-off value of 0.16 that included 2 SD (mean = 0.07, 2 SD = 0.09). When we included 3 SD, the cut-off value was 0.20 (mean = 0.07, 3 SD = 0.13). For IgG (**Figure 1B**), we obtained a cut-off value of 0.12 that included 2 SD (mean = 0.06 2 SD = 0.06). When we added 3 SD, the cut-off value was 0.15 (mean = 0.06, 3 SD = 0.09). For both IgM and IgG, few samples showed a value above the mean when 2 SD (2 and 4 samples, respectively) and 3 SD (1 sample for each isotype) were used, suggesting a high specificity (**Figures 1A, B**).

Considering individuals with no history of SARS-CoV-2 and individuals with COVID-19, the sensitivity calculated for IgM was 84.1%, the specificity was 96.7%, and the cut-off value was set at 0.1 (**Figure 1C**). For IgG, the sensitivity calculated was 100.0%, the specificity was 96.0%, and the cut-off value was set at 0.2 (**Figure 1D**).

### 3.3 Correlation between Ct values and IP for IgM and IgG isotypes

There was no significant correlation between the Ct values and the IP, neither for the IgM (R = 0.0754, p = 0.6976) nor the IgG (R = 0.1946, p = 0.3118) antibodies during the infection (**Supplement 1**).

### 3.4 Longitudinal follow-up of IgM and IgG anti-SARS-CoV-2 antibody response

The IP for IgM was affected only by group type (p = 0.0001) and clinical spectrum of disease (p < 0.0001) as well as by their interaction with time (time x group type: p = 0.0091; time x clinical spectrum of disease: p = 0.0305). The other characteristics of the population analyzed did not have significant effects (**Supplement 2**).

**Figure 2:**
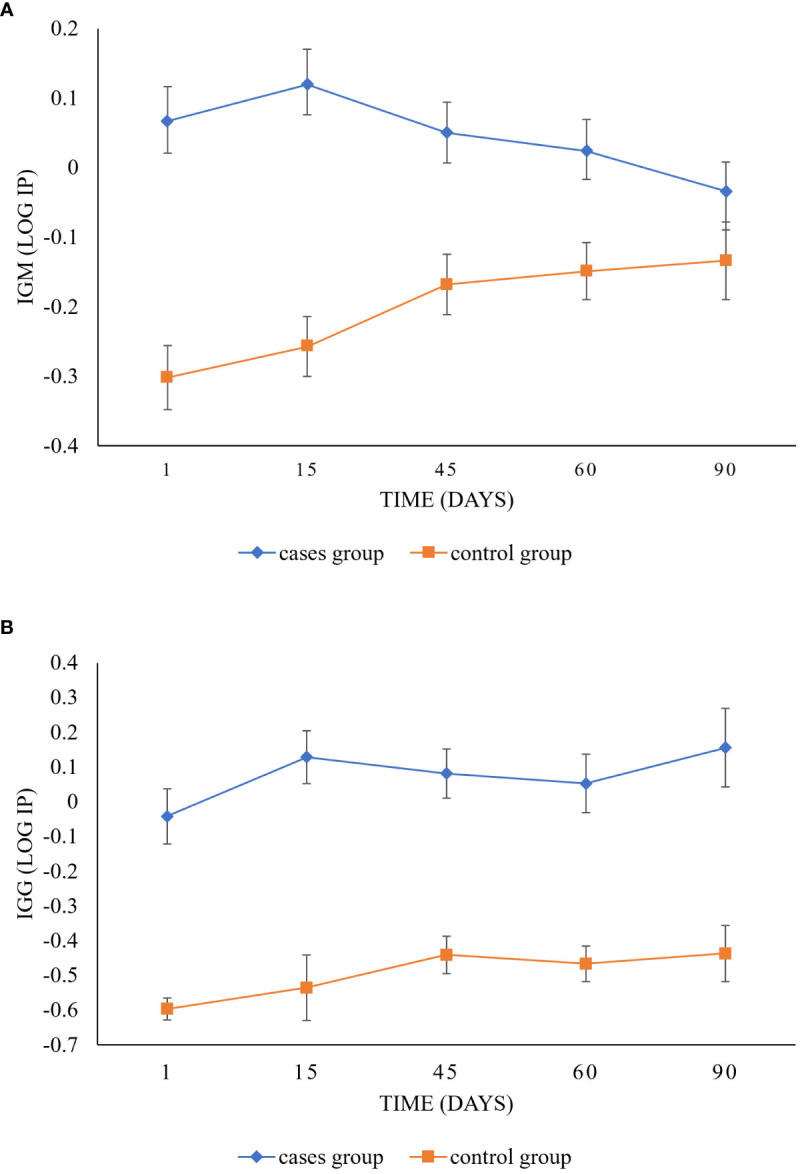
Longitudinal follow-up of IgM and IgG anti-SARS-CoV-2 antibody response between cases and control groups. The graphic shows differences between the IP values of the case group (diamond) and the control group (square) for IgM **(A)** and IgG **(B)** isotypes over time (day 1 to day 90). Significant differences can be observed between groups in IgM (p = 0.0001) and IgG (p < 0.0001). In IgM, the case group varies significantly from the control group over time (p = 0.0091). The case group showed its highest IP values between day one and day 15 and tended to decrease over time, while the control group increased its values. However, the case group never reached values above the cut-off value throughout the study. In IgG, the cases and control groups tended to increase (p = 0.0318).

As for group type, the case group showed its highest IP values between day one and day 15 and tended to decrease over time. It appeared early after the molecular diagnosis of the infection and peaked 15 days after the diagnosis but decreased for up to 45 days and continued decreasing until 90 days when the IP was well below the cut-off point (**Figure 2A**). The control group increased its values over time. However, this group never reached values above the cut-off value throughout the study (**Figure 2A**).

**Figure 3:**
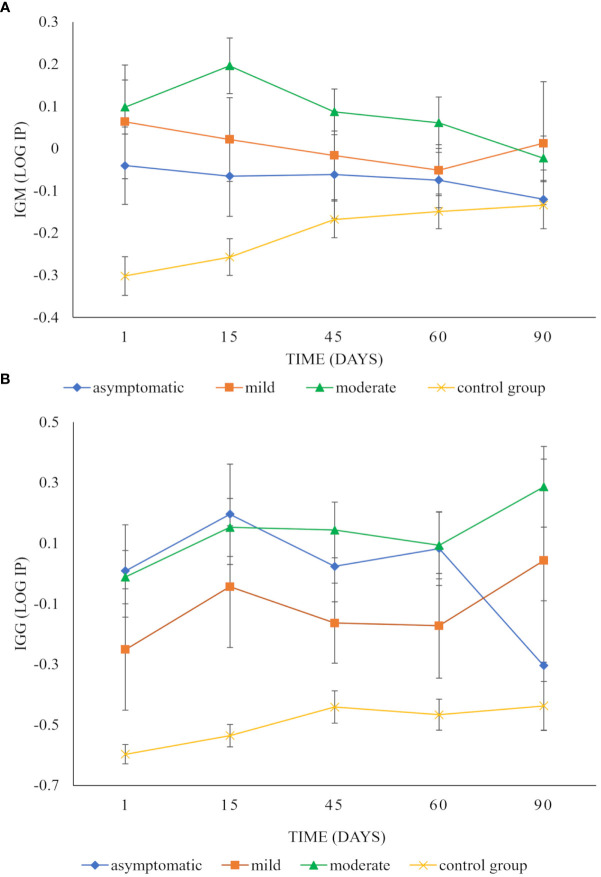
Longitudinal follow-up of IgM and IgG anti-SARS-CoV-2 antibody response in patients with different clinical spectrum of COVID-19 disease. The graphic shows differences over time between the IP values of IgM **(A)** and IgG **(B)** isotypes of individuals with mild disease (square), with moderate disease (triangle), with asymptomatic infection (diamond), and (x) with the control group. Statistical differences were observed between the clinical spectrum of disease IgM (p < 0.0001) and IgG isotypes (p < 0.0001). For IgM, individuals with moderate disease presented the highest levels of IgM at 15 days. However, their values remained above the cut-off value up to 60 days after infection. In individuals with mild disease, their highest value was observed at the time of infection, with a tendency to decrease over time. The asymptomatic individuals and the control group always showed lower values **(A)**. However, only individuals with moderate disease and the control group presented significant differences (p = 0.0021), as well as their interaction over time (p = 0.0305). For IgG, the IP values were higher for asymptomatic individuals and individuals with mild and moderate disease in contrast to the control group **(B)**. The asymptomatic individuals responded quickly to the infection and reached their maximum value in 15 days. However, at 90 days, their values were well below the cut-off value **(B)**. The individuals with moderate disease showed their maximum value 90 days post-infection. Individuals with mild disease reached their maximum on day 90. For IgG, the IP values were statistically highest in asymptomatic individuals and individuals with mild and moderate disease in contrast to the control group (p=0.0126, p=0.0264, and p<0.0001 respectively).

As for the clinical spectrum of the disease, individuals with moderate disease presented the highest levels of IgM at 15 days; however, their values remained above the cut-off value up to 60 days after infection. For individuals with mild disease, their highest value was observed at the time of infection, above the cut-off value, with a tendency to decrease over time. In contrast, the asymptomatic individuals and the control group always showed values below the cut-off (**Figure 3A**). However, significant differences were only detected between the individuals with moderate disease and the control group (p=0.0021; **Table 2**).

**Table 2:** P values as results of multiple comparisons between the clinical spectrum of disease and the control group for IgM.

	Asymptomatic	Mild	Moderate	Control
**Asymptomatic**	–	0.7479	0.2701	0.7310
**Mild**		–	0.2523	0.2822
**Moderate**			–	***0.0021***
**Control**				–

Values in bold italics are P values for significant pairs only.

The IP for IgG was affected only by group type (p < 0.0001) and clinical spectrum of disease (p < 0.0001) as well as by time (group type: p = 0.0318; clinical spectrum of disease: p = 0.0228). The other study population characteristics had no significant effects on the IP and no interactions with time (**Supplement 3**).

As for group type, the IP in the cases tended to increase over time, reaching a peak at three months after infection (**Figure 2B**). In this case, the control group followed the same trend as the case group; however, all their values were below the cut-off value (**Figure 2B**).

As for the clinical spectrum of the disease, the IP values were higher for asymptomatic individuals and individuals with mild and moderate disease, compared to the control group (**Figure 3B**). The asymptomatic individuals responded quickly to the infection and reached their maximum value in 15 days. However, at 90 days, their values were well below the cut-off value (**Figure 3B**). The individuals with moderate disease were slightly below the cut-off value at the beginning of the infection. Later, they increased to reach their maximum value 90 days post-infection. The individuals with mild disease had IP values below the cut-off point for up to 60 days. On day 90, they reached their highest value above the cut-off value (**Figure 3B**). Significant differences were detected between asymptomatic (p=0.0126), mild (p=0.0264), and moderate disease (p<0.0001) with the control group for IgG (**Table 3**).

**Table 3:** P values as results of multiple comparisons between the clinical spectrum of disease and the control group for IgG.

	Asymptomatic	Mild	Moderate	Control
**Asymptomatic**	–	0.8793	0.0572	***0.0126***
**Mild**		–	0.1663	***0.0264***
**Moderate**			–	***<0.0001***
**Control**				–

Values in bold italics are P values for significant pairs only.

### 3.5 Relationship between IgM and IgG anti-SARS-CoV-2 antibody response and characteristics of the population

**Figure 4:**
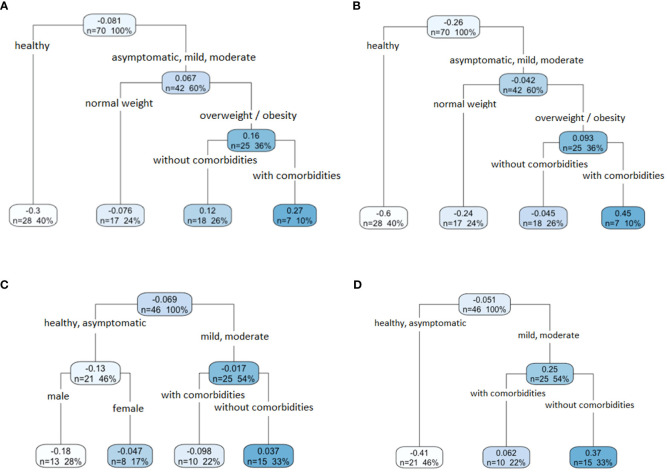
The relationship between IgM and IgG anti-SARS-CoV-2 antibody response and the characteristics of the population. A regression tree with ANOVA approximation was performed for both IgM and IgG isotypes for day one and day 90. In the case of IgM **(A)** and IgG **(B)** on day 1, asymptomatic volunteers and volunteers with mild or moderate symptoms, also overweight or obese and with comorbidities recorded the highest IP values (dark blue box). In the case of IgM **(C)** and IgG **(D)** on day 90, the volunteers with mild or moderate disease, without comorbidities, registered the highest IP values (dark blue box).

On day 1, individuals infected with SARS-CoV-2, whether asymptomatic, with mild or moderate disease, but with normal weight, had IP values below the cut-off points for both IgM (-0.076, **Figure 4A**) and IgG (-0.24, **Figure 4B**). These individuals represented 24% of the volunteers. In contrast, infected individuals, whether asymptomatic, with mild or moderate disease, but overweight or obese, and with other comorbidities, had the highest IP values for both IgM (0.27, **Figure 4A**) and IgG (0.45, **Figure 4B**). These individuals represented 10% of the volunteers.

On day 90, in the case of IgM (**Figure 4C**), the healthy individuals and those infected with the SARS-CoV-2 virus with asymptomatic infection (-0.13), and those who presented mild or moderate disease, but had additional comorbidities (-0.098), showed IP values below the cut-off point. In the case of IgG (**Figure 4D**), the healthy and asymptomatic infected individuals showed the lowest IP values (-0.41), in contrast to those patients who developed a mild or moderate outcome of the disease (0.25). In both antibodies, IgM and IgG, individuals with mild or moderate disease without comorbidities had higher IP values (0.37 and 0.37, respectively). This condition was present in 33% of the total volunteers analyzed.

## 4 Discussion

In this work, we report the detection of the anti-SARS-CoV-2 IgM and IgG antibody response, by immunoassay approach, shortly after the beginning of the symptoms and up to 90 days afterward in patients with COVID-19 detected by RT-qPCR that did not require hospitalization (cases group), and clinically healthy individuals that were not suspected of being infected with COVID-19 and with a negative RT-qPCR for SARS-CoV-2, coming from Mexico City and the State of Mexico. Although the immunoassay employed does not allow us to determine the functional nature of antibodies, we can assume from previous reports that the receptor binding domain (RBD) of the Spike protein examined for seroconversion is the target of the neutralizing antibodies against SARS-CoV-2 (Kim et al., 2021; Maciola et al., 2022; Chen et al., 2023; Yang and Du, 2021). In previous studies, the estimates of seroconversion for the RBD Spike have ranged from 91% to 99%. IgG and IgM were reported to be produced simultaneously in infected individuals (Gudbjartsson et al., 2020; Dan et al., 2021; Petrelli et al., 2022; Fekry et al., 2023).

In this study, we did not find any relationship between levels of IgM and IgG and the Ct values. The Ct value is inversely related to the viral load, and every ~3.3 increase in the Ct value reflects a 10-fold reduction in starting material (Cruz-Rangel et al., 2022). However, other studies reported that levels of IgG and IgM response could be driven by the viral load (Long et al., 2020; Masiá et al., 2021; Young et al., 2021), but these contrasting results can be explained by the heterogeneity of the studies related to the different characteristics of the study population and/or the methodology used to quantify SARS-CoV-2 and the quality of the sampling (Codina et al., 2021).

When we compared the dynamics of IgM and IgG antibodies, we observed that in the case group, the IgM appeared early after infection with SARS-CoV-2 and subsequently decreased until reaching levels below the cutoff point (> 90 days follow-up). On the other hand, the IgG antibodies began to increase early after the appearance of symptoms, and they increased steadily over time, reaching their highest levels on day 90. These data agree with other studies in which IgM appears at the early stages of the immune response and declines dramatically once IgG rises and remains for a longer time (Poland et al., 2020; Seow et al., 2020; Glück et al., 2021; Yamayoshi et al., 2021). However, Adams et al. (2020) found that IgG titers increased during the first three weeks and began to decrease eight weeks after the onset of symptoms in COVID-19 patients. In contrast, we found that IgG levels persisted over the cut-offline of 90 days after infection and persisted in a short group of patients of this same cohort after one year (data not shown).

In addition, we detect cases with subclinical and asymptomatic infections, which are of epidemiological importance for the transmission of COVID-19. We also found that antibody levels varied according to the clinical spectrum of the disease. As for the immune response dynamics, Long et al. (2020) reported that the IgG antibody response was very short in a group of asymptomatic patients with confirmed SARS-CoV-2 virus infection. Moreover, the immune response was transient and lasted only during the acute phase of the disease. Our results showed that the IgG antibodies in asymptomatic patients had the highest IP values after 15 days of infection once they had a negative RT- qPCR test. Subsequently, their levels decreased rapidly up to 45 days post-infection. This suggests that the humoral immunity against SARS-CoV-2 may not be long-lasting in people who have experienced an asymptomatic infection. Other studies have also analyzed the response to mild and moderate COVID-19 disease. Lu et al. (2020) described the dynamics of IgG isotypes in response to SARS-CoV-2 infection. They found that IgG in individuals with mild and moderate symptoms appeared on day seven after the onset of symptoms, reached a maximum peak at 28 days, decreased around 35 days, and remained stable after 40 days. In contrast, we separately analyzed the anti-SARS-CoV-2 antibody response in individuals with mild and moderate disease. In the case of individuals with moderate disease, IgG titers were highest around 15 days after infection, and their levels remained high up to 90 days after symptoms onset. In the case of patients with mild disease, IgG titers were under the cut-off during almost all the follow-up and reached their maximum value 90 days after infection. This suggests that the SARS-CoV-2 infection in patients with mild symptoms and asymptomatic patients is more likely controlled by the innate immune response, which acts in anticipation of the adaptive response. Although we found different dynamics of the production of IgG antibodies in individuals with mild, moderate, and asymptomatic disease, no significant differences in antibody values over time concerning disease severity were observed. However, we found differences in IgM titers between individuals with moderate disease and asymptomatic individuals, suggesting that the higher intensity and duration of the IgM response is related to the clinical spectrum of disease (Long et al., 2020; Zhao et al., 2020; Liu et al., 2021).

Furthermore, through regression trees, it was found that factors such as the clinical spectrum disease, BMI, and comorbidities are related to IgM and IgG antibodies, which could determine immune response development. We observed that at the beginning of the infection, the asymptomatic, mild, and moderate cases fell into a single group for both, IgM and IgG. However, 90 days after the infection, the asymptomatic individuals grouped with the control group, suggesting that the asymptomatic individuals lost the antibodies elicited by the infection quickly. The COVID-19 patients with overweight and obesity and other comorbidities developed higher levels of IgM and IgG at the beginning of the infection. It is commonly thought that immunological changes in obesity affect humoral immunity, mainly by decreasing the secretion of antibodies (De Heredia et al., 2012). However, we found that IgM and IgG antibodies are produced very early after the infection in overweight and obese patients, in contrast to normal-weight patients. This behavior could be related to overstimulation of the immune system in obese or overweight patients. These findings agree with findings reported in other studies. For example, Racine-Brzostek et al. (2021) found that patients with obesity (BMI > 30 kg/m2) had higher anti-SARS-CoV-2 antibody levels than lean patients (BMI < 25 kg/m2). Patients with obesity and non-severe disease courses presented higher levels of neutralizing antibodies compared to their counterparts with normal weight (Petersen et al., 2021; Racine-Brzostek et al., 2021; Belchior-Bezerra et al., 2022).

In addition, at the end of follow-up, the highest values were associated with individuals with mild or moderate disease without comorbidities. These findings suggest that the presence of comorbidities compromises the duration of the immune response, causing the antibodies to have a short lifetime and accelerating the decrease of IgG. In addition, the duration of the response in healthy people was observed to be longer. This is an outstanding result since a large part of the population in Mexico has one or more comorbidities such as diabetes mellitus, hypertension, and some coronary disease that make them susceptible to rapidly losing the protection given by the SARS-CoV-2 infection. This aligns with Jing et al. (2022), who found that IgG decreased between 35- and 70-days post-infection in individuals with comorbidities. This behavior in the immune response must be studied in more detail to determine how these conditions of overweight and obesity and other chronic diseases could be related to the levels of anti-SARS-CoV-2 antibodies. In addition, the severity of the disease could be responsible for the duration of IgM and IgG antibodies (Long et al., 2020; Zhao et al., 2020).

It is necessary to point out that the sample size is a limitation of our work since it reduces the generalization of the findings in a larger population. Hence, the results must be interpreted with caution. Although these limitations may cause bias problems, this work provides important information about the dynamics of the antibody response to anti-SARS-CoV-2 in acute and convalescent volunteers.

In conclusion, we report the dynamics of the antibody response in patients infected with SARS-CoV-2 who did not require hospitalization in a longitudinal study of 90 days. Although our study has some limitations, which were pointed out previously, our results suggested that the level, duration, and dynamics of the antibody response in these patients are related to the clinical spectrum of disease, as well as to BMI and the presence/absence of comorbidities. However, further studies with a larger sample size are needed to confirm this assertion. Obesity and other comorbidities are major risk factors for disease severity and death and are associated with an inflammatory state affecting immune function. Nevertheless, it remains unclear whether these conditions are due to discrepancies in medical care or some other unveiled mechanisms that predispose the infected patients to develop severe or long-lasting symptoms of COVID-19 and whether the inflammatory state is involved. The reasonable approach to fill these knowledge gaps is the study of large cohorts of infected individuals, the simultaneous analysis of all branches of the immune response, and the correlation with the severity of symptoms, comorbidities, and genetic predisposition. COVID-19 is an extraordinarily complex disease, and each patient is a different expression of the pathogenic potential of this virus, which we still need to understand.

## Data availability statement

The raw data supporting the conclusions of this article will be made available by the authors, without undue reservation.

## Ethics statement

The studies involving humans were approved by Scientific and Ethics Committee of the Medical School of the Universidad Nacional Autónoma de México (UNAM). The studies were conducted in accordance with the local legislation and institutional requirements. Written informed consent for participation in this study was provided by the participants’ legal guardians/next of kin.

## Author contributions

HPJ, ASV, and HGA: Conceptualization, cohort follow-up, methodology, formal analysis, investigation, writing – original draft and prepared the figures and tables. EGR, LRV, and PM: investigation, cohort follow-up, methodology, writing – review, and data curation. TP, MR: visualization, cohort recruitment, and database. EH, MAP, MEZ: literature review, sample organization, and collected the dataset. CL, MM: taking samples and determining the Ct values. BT: data analysis and writing - review. LAP, SL: validation, resources, writing review, and editing. AA, CFA, and CX: conceptualization, project administration, supervision, writing- review and editing, funding acquisition, and resources. All authors contributed to the article and approved the submitted version.
