# Supplementary material for: Longitudinal anti-SARS-CoV-2 antibody immune response in acute and convalescent patients
Source: Front Cell Infect Microbiol. 2023 Sep 8;13:1239700. doi: 10.3389/fcimb.2023.1239700 (PMC10515199; doi:10.3389/fcimb.2023.1239700)
Supplement: Supplementary file 1 [file Table_1.docx]

Supplementary Material

**Longitudinal anti-SARS-CoV-2 antibody immune response in acute and convalescent patients**

**Horacio Pérez-Juárez, Angélica Serrano-Vázquez, Héctor Godínez-Álvarez, Enrique González, Liliana Rojas-Velázquez, Patricia Moran, Tobías Portillo, Manuel Ramiro, Eric Hernández, Ma. Ángeles Padilla, Martha E. Zaragoza, Blanca Taboada, Laura A. Palomares, Susana López, Alejandro Alagón*, Carlos F. Arias**, Cecilia Ximénez*****

*** Correspondence authors:**

*[alejandro.alagon@ibt.unam.mx](mailto:alejandro.alagon@ibt.unam.mx) (AA)**[arias@ibt.unam.mx](mailto:arias@ibt.unam.mx) (CFA), ***[cximenez2005@yahoo.com.mx](mailto:cximenez2005@yahoo.com.mx) (CX).

# Supplementary Tables

**Supplementary 1.** Correlation analysis between Log IP and Ct values.

with * had p ≤ 0.05.

|  | **CT** | **CV** | **IP IgG** | **IP IgM** |
| --- | --- | --- | --- | --- |
| CT | 1.0000 | -0.9513* | 0.1946 | 0.0754 |
| CV | -0.9513* | 1.0000 | -0.2011 | -0.0987 |
| IP IgG | 0.1946 | -0.2011 | 1.0000 | 0.4806 |
| IP IgM | 0.0754 | -0.0987 | 0.4806 | 1.0000 |

**Supplementary 2.** Results of the repeated measures ANOVA were conducted to evaluate the effects of the study population characteristics and time on the IP for IgM of the cases and control groups. Group type: case and control; severity of disease: asymptomatic, mild, and moderate; sex: male and female; age: ˂ 60 and ≥ 60; comorbidities: with or without comorbidities; BMI: ≤ 25 and ˃25.

| FACTOR | F | d.f. | P |  |
| --- | --- | --- | --- | --- |
| GROUP TYPE | | | |  |
| *Within-subjects effects* |  | | |  |
| Time | 1.0334 | 4,37 | 0.403 |  |
| Time x group | 3.9454 | 4,37 | 0.0091 |  |
| *Between-subjects effect* |  | | |  |
| Group | 17.9334 | 1,40 | 0.0001 |  |
| CLINICAL SPECTRUM OF DISEASE | | | |  |
| *Within-subjects effects* |  | | |  |
| Time | 0.9218 | 4,35 | 0.4623 |  |
| Time x clinical spectrum of disease | 2.0226 | 12,93 | 0.0305 |  |
| *Between-subjects effect* |  |  |  |  |
| Cliinical spectrum of disease | 9.3614 | 3,38 | ˂0.0001 |  |
| SEX | | | |  |
| *Within-subjects effects* |  | | |  |
| Time | 0.8897 | 4,35 | 0.4803 |  |
| Time x group | 3.771 | 4,35 | 0.0119 |  |
| Time x sex | 0.8 | 4,35 | 0.5334 |  |
| Time x group x sex | 0.2688 | 4,35 | 0.8961 |  |
| *Between-subjects effect* |  | | |  |
| Group | 15.0714 | 1,38 | 0.0004 |  |
| Sex | 0.4195 | 1,38 | 0.5211 |  |
| Group x sex | 1.3926 | 1,38 | 0.2453 |  |
| AGE | | | |  |
| *Withing-subjectt effects* |  | | |  |
| Time | 1.9584 | 4,35 | 0.1225 |  |
| Time x group | 1.4146 | 4,35 | 0.2495 |  |
| Time x age | 1.2142 | 4,35 | 0.3223 |  |
| Time x group x age | 1.2578 | 4,35 | 0.305 |  |
| *Between-subjects effect* |  | | |  |
| Group | 13.2971 | 1,38 | 0.0008 |  |
| Age | 0.1962 | 1,38 | 0.6603 |  |
| Group x age | 1.0725 | 1,38 | 0.3069 |  |
| COMORBIDITIES | | | |  |
| *Withing-subjectt effects* |  |  |  |  |
| Time | 1.2479 | 4,35 | 0.3089 |  |
| Time x group | 2.2194 | 4,35 | 0.0869 |  |
| Time x comorbidities | 0.7578 | 4,35 | 0.5598 |  |
| Time x group x comorbidities | 1.2665 | 4,35 | 0.3016 |  |
| *Between-subjects effect* |  |  |  |  |
| Group | 8.2911 | 1,38 | 0.0065 |  |
| Comorbidities | 1.0184 | 1,38 | 0.3193 |  |
| Group x comorbidities | 0.1058 | 1,38 | 0.7468 |  |
| BODY MASS INDEX (BMI) | | | | |
| *Withing-subjectt effects* |  |  |  |  |
| Time | 1.0637 | 4,35 | 0.389 |  |
| Time x group | 3.9235 | 4,35 | 0.0098 |  |
| Time x BMI | 1.1846 | 4,35 | 0.3346 |  |
| Time x group x BMI | 1.0477 | 4,35 | 0.3068 |  |
| *Between-subjects effect* |  |  |  |  |
| Group | 17.2432 | 1,38 | 0.0002 |  |
| BMI | 0.4528 | 1,38 | 0.5051 |  |
| Group x BMI | 0.8581 | 1,38 | 0.3601 |  |

**Supplementary 3.** Results of the repeated measures ANOVA were conducted to evaluate the effects of the study population characteristics and time on the IP for IgG in the cases and control groups. Group type: case and control; severity of disease: asymptomatic, mild, and moderate; sex: male and female; age: ˂ 60 and ≥ 60; comorbidities: with or without comorbidities; BMI: ≤ 25 and ˃25.

| FACTOR | F | d.f. | P |
| --- | --- | --- | --- |
| GROUP TYPE | | | |
| *Withing-subjectt effects* |  | | |
| Time | 2.9708 | 4,37 | 0.0318 |
| Time x group | 0.891 | 4,37 | 0.479 |
| *Between-subjects effect* |  | | |
| Group | 38.3782 | 1,40 | ˂0.0001 |
| CLINICAL SPECTRUM OF DISEASE | | | |
| *Withing-subjectt effects* |  |  | |
| Time | 3.2511 | 4,35 | 0.0228 |
| Time x clinical spectrum of disease | 1.2899 | 12,92.893 | 0.2377 |
| *Between-subjects effect* |  | | |
| Clinical spectrum of disease | 15.4318 | 3,38 | ˂0.0001 |
| SEX | | | |
| *Withing-subjectt effects* |  | | |
| Time | 2.5705 | 4,35 | 0.0549 |
| Time x group | 0.7731 | 4,35 | 0.5501 |
| Time x sex | 0.0787 | 4,35 | 0.9883 |
| Time x group x sex | 0.983 | 4,35 | 0.4295 |
| *Between-subjects effect* |  | | |
| Group | 32.2619 | 1,38 | ˂0.0001 |
| Sex | 0.3192 | 1,38 | 0.5754 |
| Group x sex | 0.0033 | 1,38 | 0.9544 |
| AGE | | | |
| *Withing-subjectt effects* |  | | |
| Time | 1.4245 | 4,35 | 0.2463 |
| Time x group | 1.4245 | 4,35 | 0.2463 |
| Time x age | 0.1344 | 4,35 | 0.9686 |
| Time x group x age | 0.4085 | 4,35 | 0.8013 |
| *Between-subjects effect* |  |  | |
| Group | 25.7434 | 1,38 | ˂0.0001 |
| Age | 1.1122 | 1,38 | 0.2983 |
| Group x age | 1.0143 | 1,38 | 0.3202 |
| COMORBIDITIES | | | |
| *Withing-subjectt effects* |  | | |
| Time | 0.7697 | 4,35 | 0.5523 |
| Time x group | 0.3395 | 4,35 | 0.8495 |
| Time x comorbidities | 0.1274 | 4,35 | 0.9715 |
| Time x group x comorbidities | 0.03 | 4,35 | 0.9982 |
| *Between-subjects effect* |  | | |
| Group | 15.9056 | 1,38 | 0.0003 |
| Comorbidities | 0.0005 | 1,38 | 0.9824 |
| Group x comorbidities | 0.6563 | 1,38 | 0.4229 |
| BODY MASS INDEX (BMI) | | | |
| *Withing-subjectt effects* |  | | |
| Time | 2.9371 | 4,35 | 0.0341 |
| Time x group | 0.9026 | 4,35 | 0.473 |
| Time x BMI | 0.4726 | 4,35 | 0.7555 |
| Time x group x BMI | 0.8676 | 4,35 | 0.493 |
| *Between-subjects effect* |  | | |
| Group | 36.531 | 1,38 | ˂0.0001 |
| BMI | 0.2278 | 1,38 | 0.6359 |
| Group x BMI | 0.2655 | 1,38 | 0.6093 |
